# Can K-ras Gene Mutation Be Utilized as Prognostic Biomarker for Colorectal Cancer Patients Receiving Chemotherapy? A Meta-Analysis and Systematic Review

**DOI:** 10.1371/journal.pone.0077901

**Published:** 2013-10-21

**Authors:** Yuan-Yi Rui, Dan Zhang, Zong-Guang Zhou, Cun Wang, Lie Yang, Yong-Yang Yu, Hai-Ning Chen

**Affiliations:** 1 Department of Gastrointestinal Surgery, West China Hospital, Sichuan University, Chengdu, Sichuan, China; 2 Institute of Digestive Surgery, West China Hospital, Sichuan University, Chengdu, Sichuan, China; Technische Universität Dresden, Medical Faculty, Germany

## Abstract

**Introduction:**

K-ras gene mutations were common in colorectal patients, but their relationship with prognosis was unclear.

**Objective:**

Verify prognostic differences between patient with and without mutant K-ras genes by reviewing the published evidence.

**Method:**

Systematic reviews and data bases were searched for cohort/case-control studies of prognosis of colorectal cancer patients with detected K-ras mutations versus those without mutant K-ras genes, both of whom received chemotherapy. Number of patients, regimens of chemotherapy, and short-term or long-term survival rate (disease-free or overall) were extracted. Quality of studies was also evaluated.

**Principal Findings:**

7 studies of comparisons with a control group were identified. No association between K-ras gene status with neither short-term disease free-survival (OR=1.01, 95% CI, 0.73-1.38, P=0.97) nor overall survival (OR=1.06, 95% CI, 0.82-1.36, P=0.66) in CRC patients who received chemotherapy was indicated. Comparison of long-term survival between two groups also indicated no significant difference after heterogeneity was eliminated (OR=1.09, 95% CI, 0.85-1.40, P=0.49).

**Conclusions:**

K-ras gene mutations may not be a prognostic index for colorectal cancer patients who received chemotherapy.

## Introduction

Colorectal cancer (CRC) is one of the most important public health problems. Its prevalence was the third in North America and Europe and the fifth in Asia (also fifth in China) among the malignant diseases [1-3]. Despite the decline of the disease prevalence in some developed countries, colorectal cancer remains a fatal disease throughout the rest of the world. Clinicians have reached a consensus that treatment of CRC should be a comprehensive project, which consists of surgery, adjuvant chemotherapy and certain targeted therapy. Presently, 5-FU based chemotherapy has been recognized as first line regimen and utilized as adjuvant (also neo-adjuvant) treatment of CRC patients [4,5].

The Kirsten ras (K-*ras*) gene is one member of the ras gene family (H-, K- and N-ras) which encodes highly similar membrane-localized G proteins with molecular weight of 21 kDa [6]. All of the three different proteins are capable of binding and hydrolyzing GTP and participate in a signal transmission pathway from cytoplasm to nucleus [7]. Ras gene family control multiple pathways affecting cell growth, differentiation and apoptosis by interacting with a series of coordinators and effectors, thus they were recognized as key targets in tumor pathogenesis. The incidence of *ras* mutation varies strongly among different human tumors. In particular, oncogenic KRAS mutations are detected in approximately 20-50% of primary colorectal tumors [8-10].

Although recent meta-analysis and a multi-center study reported no association between K-ras mutations and CRC patients’ prognosis could not be indicated, the largest study focused on this issue, the RASCAL collaborative study, showed there might be connections between G12V mutation and poorer prognosis [11-13]. Similar relationship was also indicated by our preliminary research, which suggested that the presence of a K-ras mutation might lead to a lower CRC survival rate [14]. Current clinical trials verified that K-ras gene mutations were related to Cetuximab resistance in mCRC (metastatic colorectal cancer) patients [15]. However, whether mutant K-ras gene affected the survival rate of CRC patients who received adjuvant chemotherapy was still in controversy. Ahnen et al reported CRC patients with wild type K-ras gene benefit significantly more than those with codon 12 mutation subtype from chemotherapy [16]. Nevertheless, results from studies in recent decade failed to repeat such a trend [17-22]. Our study aimed to apply meta-analysis to clarify whether K-ras mutation might affect the prognosis of CRC patients who received adjuvant chemotherapy and could be utilized as a predictive biomarker for chemotherapy.

## Methods

### Search strategy

We searched electronic database of PubMed, EmBase and Cochrane Library up to April, 2013. For example, the search strategy for PubMed used the strings (((("Rectal Neoplasms"[Mesh]) OR "Colorectal Neoplasms"[Mesh]) AND "Genes, ras"[Mesh]) AND "Chemotherapy, Adjuvant" [Mesh]), limited to humans. The language of all publications was limited to English only.

### Inclusion and exclusion criteria

This meta-analysis included studies reported detection of K-ras mutations in CRC patients and their prognosis (overall survival and/or disease free survival).Patients were all diagnosed with CRC, proven by biopsy, and their chemotherapy regimens should be reported.

### Quality assessment and data collection

All studies were peer-reviewed by two researchers independently. Each included study was assessed according to “The Newcastle-Ottawa Scale for assessing the quality of non-randomized studies in meta-analyses” [23]. Any disagreement in quality assessment and data collection was discussed and solved using a third senior researcher as a referee.

The general information extracted included country, publication year, sample size, general characteristics of patients, and intervention details. Data for the prognostic outcomes measures mentioned here were extracted. Data, summarized as total number and events for each group, were extracted. Chemotherapy regimens were derived from the reports, if possible.

### Statistical analysis

Outcomes of included studies were synthesized by Review Manager (Version 5.2, 2012, Copenhagen: The Nordic Cochrane Centre, The Cochrane Collaboration). The statistical method was referred to the Cochrane Handbook for Systematic Review of Interventions [24].

For pooled estimate of discontinuous data, odds ratios (ORs) were calculated by using a fixed effects model (or random effects model, if there was heterogeneity between studies) [24]. The Mantel-Haenszel test was used to test significance, with p<0.05 considered statistically significant. Heterogeneity between comparable studies was tested in all analyses using a standard chi-square test for between-study heterogeneity and considered significant at p<0.1 [24].

## Results

### Search and selection

**Figure 1:**
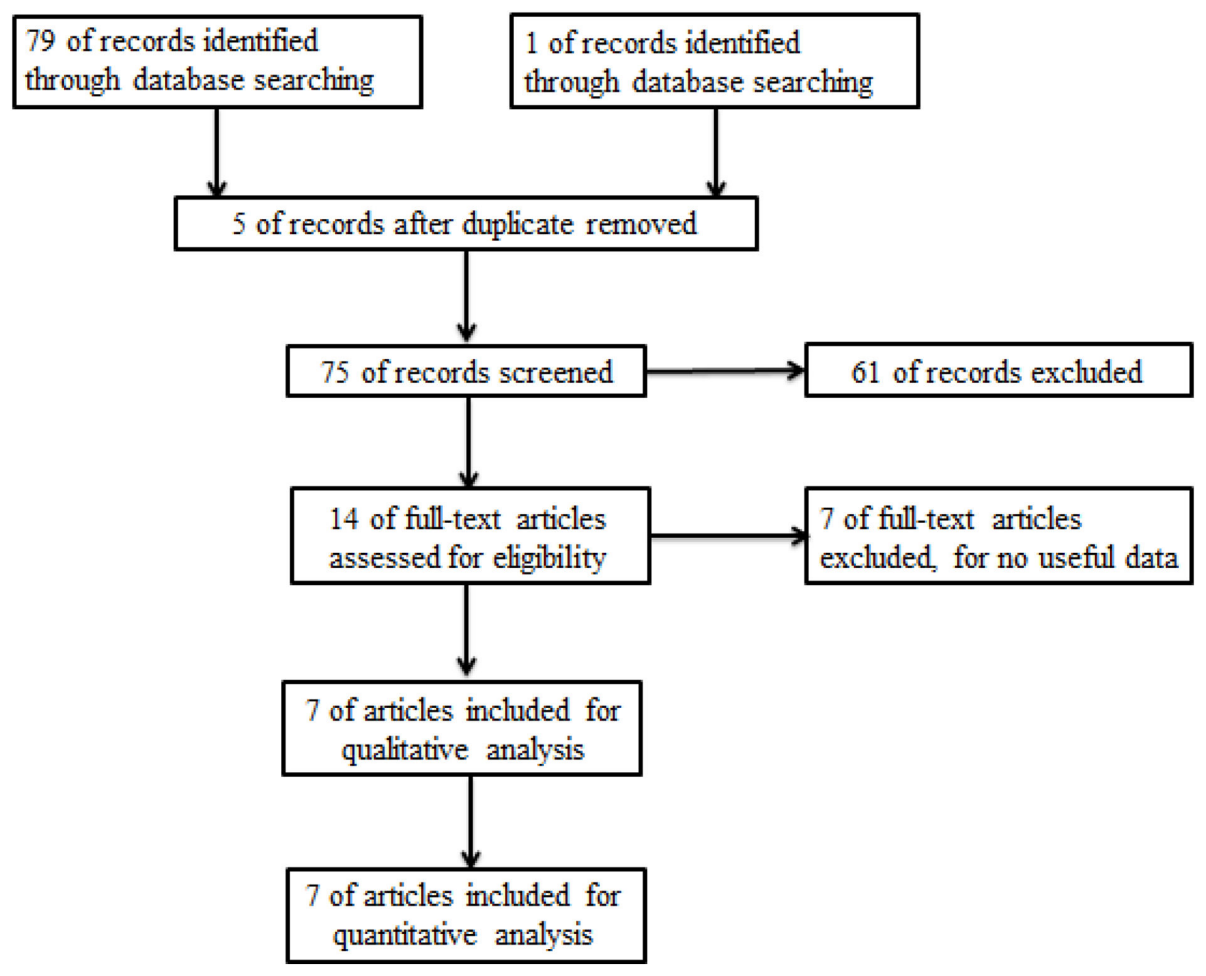
Flow Diagram.

A flow chart illustrating search and selection criteria is provided in Figure 1. 7 studies with a total number of 2334 patients (1550 with Wild type K-ras gene and 784 with mutations, all are stage II or III) were eventually included for analysis. Baseline status and quality level are listed in Table 1 [16-22].

**Table 1:** General Information of Included Studies.

**Study**	**Country**	**Participants**	**Comparison**	**Chemotherapy**	**K-ras mutation**	**Outcome Measures**	Quality*
					**detection**		
Ahnen 1998	USA	Stage II and III	wild type (n=131)	levamisole or 5-FU plus levamisole	PCR-SSCP	3-year and 5-year OS	8 stars
		CRC patients	versus mutant type (n=89)				
Bleeker 2001	Netherland	Dukes C CRC	wild type (n=40)	5-FU/lev/leuco or 5-FU/lev	DGGE	3-year and 5-year OS	8 stars
		patients	versus mutant type (n=15)				
Ogino 2009	USA	Stage III CRC	wild type (n=330)	5-FU/leucovorin or IFL	Pyrosequencing	3-year and 5-year OS	8 stars
		patients	versus mutant type (n=178)	(irinotecan, 5-FU and leucovorin)		and 2-year DFS	
Chang 2011	Korea	Stage II and III	wild type (n=51)	FL or FOLFOX	DNA-sequence	3-year OS and 2-year	8 stars
		CRC patients	versus mutant type (n=15)			DFS	
Gnanasampath-	Australia	Dukes’ C	wild type (n=290)	5-FU/levamisole or	PCR-SSCP	3-year and 5-year OS	8 stars
an 2011		patients	versus mutant type (n=138)	5-FU/leucovorin			
Hutchins 2011	UK	Stage II and III	wild type (n=524)	5-FU/folinic acid	Pyrosequencing	2-year DFS	8 stars
		CRC patients	versus mutant type (n=260)				
Sec 2012	Poland	Unclear	wild type (n=184)	Irinotecan or oxaliplatin-based	PCR-RFLP	3-year and 5-year OS	7 stars
			versus mutant type (n=89)	therapy			

* Quality of studies was assessed, according to “The Newcastle-Ottawa Scale for assessing the quality of non-randomized studies in meta-analyses” standard, by numbers of stars.

### 2-year disease-free survival rate

**Figure 2:**
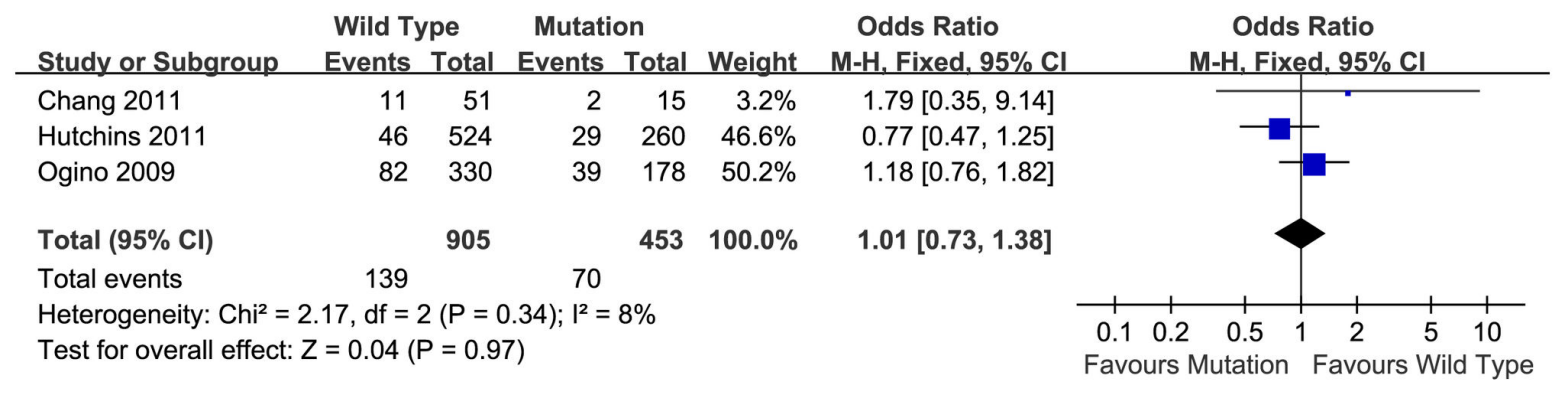
2-year disease-free survival rate of wild type or mutant K-ras gene in patients received chemotherapy.

Based on 3 studies including Caucasian and Asian people, the pooled 2-year disease-free survival rate of the K-ras wild type group was 84.6% (766 of 905), and that of mutation group was 84.5% (383/453). Meta-analysis showed no association between K-ras mutations and survival rate in patients who received chemotherapy (OR=1.01, 95% CI, 0.73-1.38, P=0.97) (Figure 2). There was no significant heterogeneity between studies (P=0.34). 

### 3-year over-all survival rate

**Figure 3:**
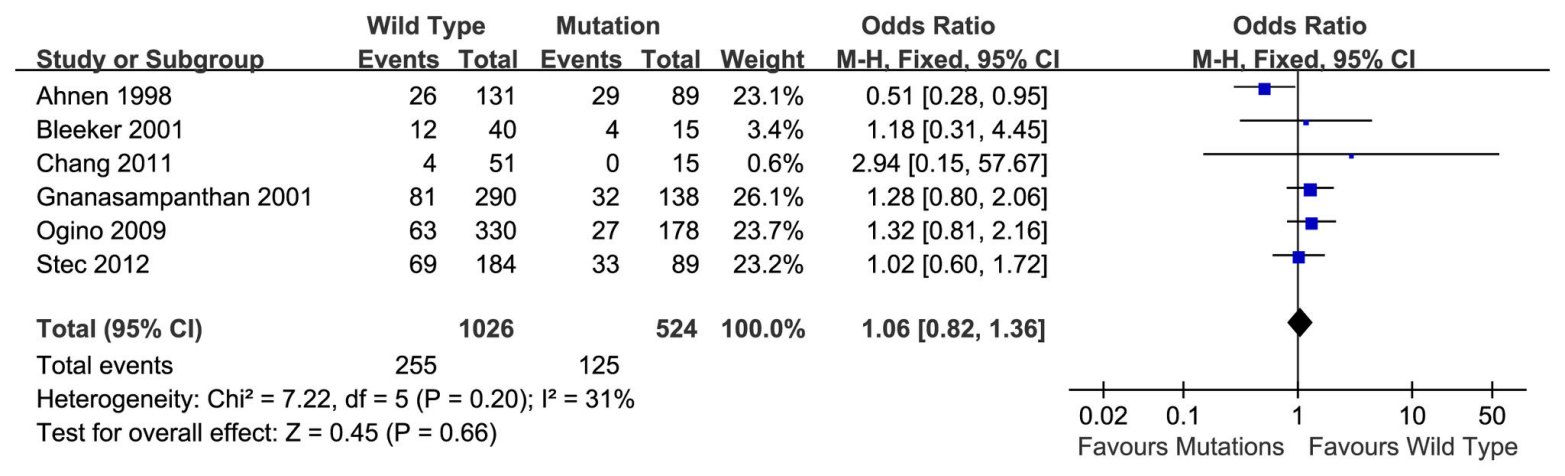
3-year overall survival rate of wild type or mutant K-ras gene in patients received chemotherapy.

Six articles reporting 3-year over-all survival rate were included. The pooled 3-year overall survival rate of patients with wild type K-ras gene was 75.1%, while that of patients with mutant K-ras gene was 76.1%. Meta-analysis also indicated no significant association between K-ras status and prognosis in patients who received chemotherapy (OR=1.06, 95% CI, 0.82-1.36, P=0.66) (Figure 3). There was no significant heterogeneity between reports (P=0.20).

### 5-year over-all survival rate

**Figure 4:**
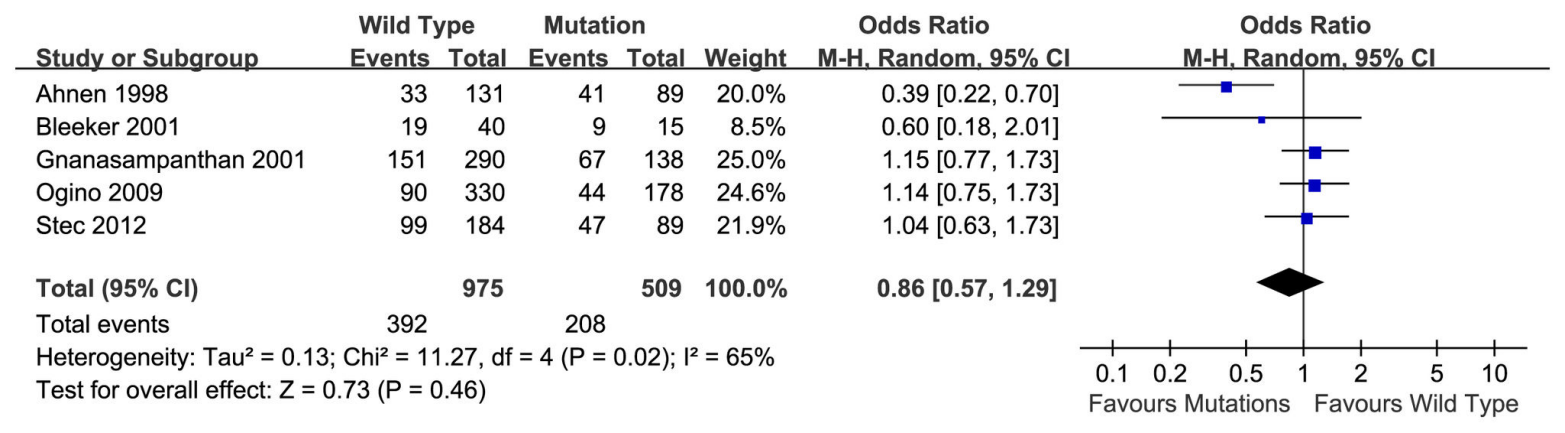
5-year overall survival rate of wild type and mutant K-ras gene in patients received chemotherapy, with Random Model.

**Figure 5:**
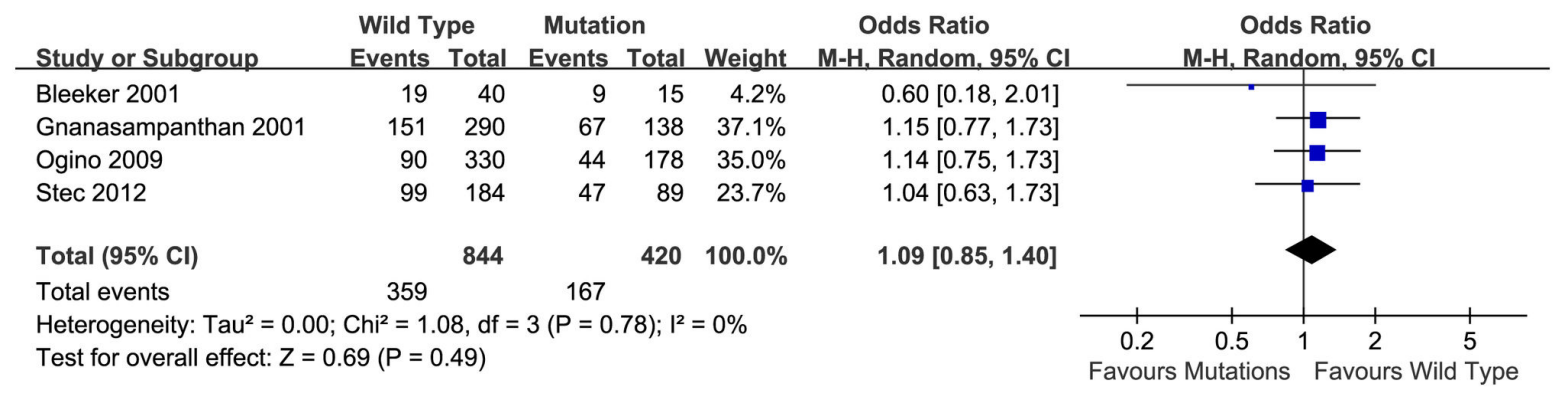
5-year disease-free survival rate of wild type and mutant K-ras gene in patients received chemotherapy after rejecting Ahnen 1998.

**Figure 6:**
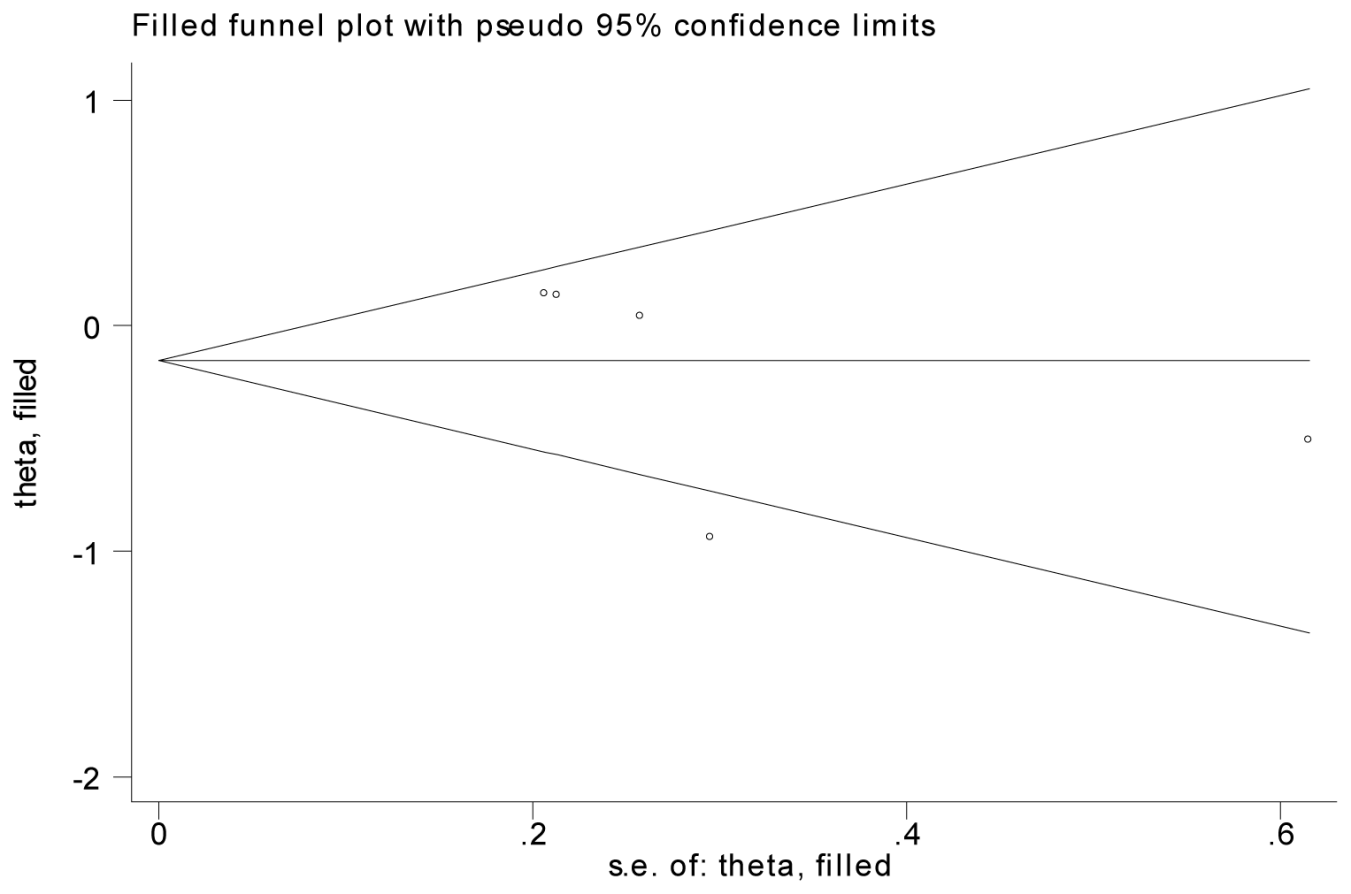
Funnel plot of 5-year DFS survival rate after fill-and trim method.

Based on 5 articles, the pooled 5-year overall survival rate of wild type K-ras gene group was 59.8%, compared to a survival rate of 59.1% in the K-ras mutant group. Meta-analysis indicated that no association between K-ras mutation and survival rate in CRC patients with chemotherapy (OR=0.86, 95% CI, 0.57-1.29, P=0.46) (Figure 4). Heterogeneity between reports was indicated by the analysis (P=0.02) and couldn’t be eliminated by changing to random model (Figure 4). We identified the report by Ahnen 1998 caused the bias. No heterogeneity was found after we excluded the certain article (P=0.78) and no significant association between K-ras mutation and prognosis, either (OR=1.09, 95% CI, 0.85-1.40, P=0.49) (Figure 5). Fill-and-trim method was used and no significant relationship was indicated (Figure 6).

## Discussion

Presently, it has come to an agreement that comprehensive therapy should be applied during the treatment of CRC, including surgery, adjuvant chemotherapy and biotherapy. 5-FU based adjuvant chemotherapy was recognized as first-line therapy and utilized worldwide in the 21st century. Meanwhile, researchers paid special attention to the mutation status of specific genes and its inference on chemotherapy. P53 mutation status was proved to be a potential biomarker in predicting prognosis for CRC patients receiving neo-adjuvant radiation-based treatment [30]. Co-relationship of MSI-H/dMMR and 5-FU based adjuvant chemotherapy was clarified by clinical trials [25]. However, the potential relationship between patients’ other biomarker status who received chemotherapy and their prognosis is still controversial. 

2^nd^ RASCAL collaborative study indicated codonG12V mutation was related to poorer long-term survival rate of CRC patients [13]. Recent studies also indicated codon 12 or 13 mutation might be a predictive mark of resistance to Cetuximab and Panitumumab [15,26-28,31,32]. However, whether these mutations could play a predictive role in CRC patients who received 5-FU based chemotherapy was still unclear. The current meta-analysis of 7 articles systematically evaluated the relationship between K-ras status and response to adjuvant chemotherapy after surgery. This study indicated that patients with wild type K-ras gene didn’t benefit further from chemotherapy. We could see that patients with or without mutant K-ras gene shared similar pooled short-time DFS and OS rate and long-term OS rate, which were not statistically significant. In other words, K-ras gene might not be considered as one of the biomarkers to predict patients’ response to current first line chemotherapy.

Since only one article included in this meta-analysis reported on the prognostic significance of specific KRAS mutations in codons 12 and 13, we were unable to further analyze the potential relationship between these specific mutations and CRC prognosis. Gnanasampathan et al reported that patients with wild type and codon 13 mutations derived significant benefit from chemotherapy, while those with codon 12 mutations got limit benefit [20]. Imamura et al also reported patients with mutant codon 12 suffered poorer cancer-specific survival rate [33]. Although we researchers anticipated discovering similar result from our study, K-ras gene subtype mutations failed to show their predictive capability. None of other 6 studies reported prognosis of CRC patients with codon 12 or 13 respectively, which led to sub-group analysis failed to be utilized. 

Since 5-FU based chemotherapy has been recommended as the first-line regimens for stage III patients (oxaliplatin is not recommended for stage II patients, while other 5-FU chemotherapies are accepted [29]), it has become a burning question that patients in which CRC stage benefit most from the standard treatment. Unfortunately, current meta-analysis can’t answer the question. Mixture of Stage II and Stage III CRC patients also affected the sensitivity of this analysis. Among the 7 included articles, only 2 articles reported patients received standard 5-FU based chemotherapy [19]. Different chemotherapy regimens also interfered with the result of our study. One article report non-standard treatment mainly depended on levamisole, while another studies reported regimen based on oxaliplatin or irinotecan, which were not standard first line treatment, according to latest NCCN guidelines [4,5,16,22]. None of these studies reported the cycles that CRC patients received. These differences might lead to clinic bias when analysis was applied.

## Conclusions

This study firstly indicates that there may be no association between K-ras mutant status and short/long term prognosis of patients who receive adjuvant chemotherapy. Our data supports that K-ras may not be a useful predictive biomarker of CRC patients’ prognosis. We expect further studies to prove the relationship between specific K-ras mutation and patient’s different outcomes. 

All authors contributed in the writing and critical development of the manuscript. All authors have read and approved the final manuscript. All authors consider it is an original article.

## Supporting Information

Checklist S1 **PRISMA checklist for meta-analysis.**
(TIF)
