# Supplementary figures and images for: Can K-ras Gene Mutation Be Utilized as Prognostic Biomarker for Colorectal Cancer Patients Receiving Chemotherapy? A Meta-Analysis and Systematic Review
Source: PLoS One. 2013 Oct 21;8(10):e77901. doi: 10.1371/journal.pone.0077901 (PMC3804628; doi:10.1371/journal.pone.0077901)

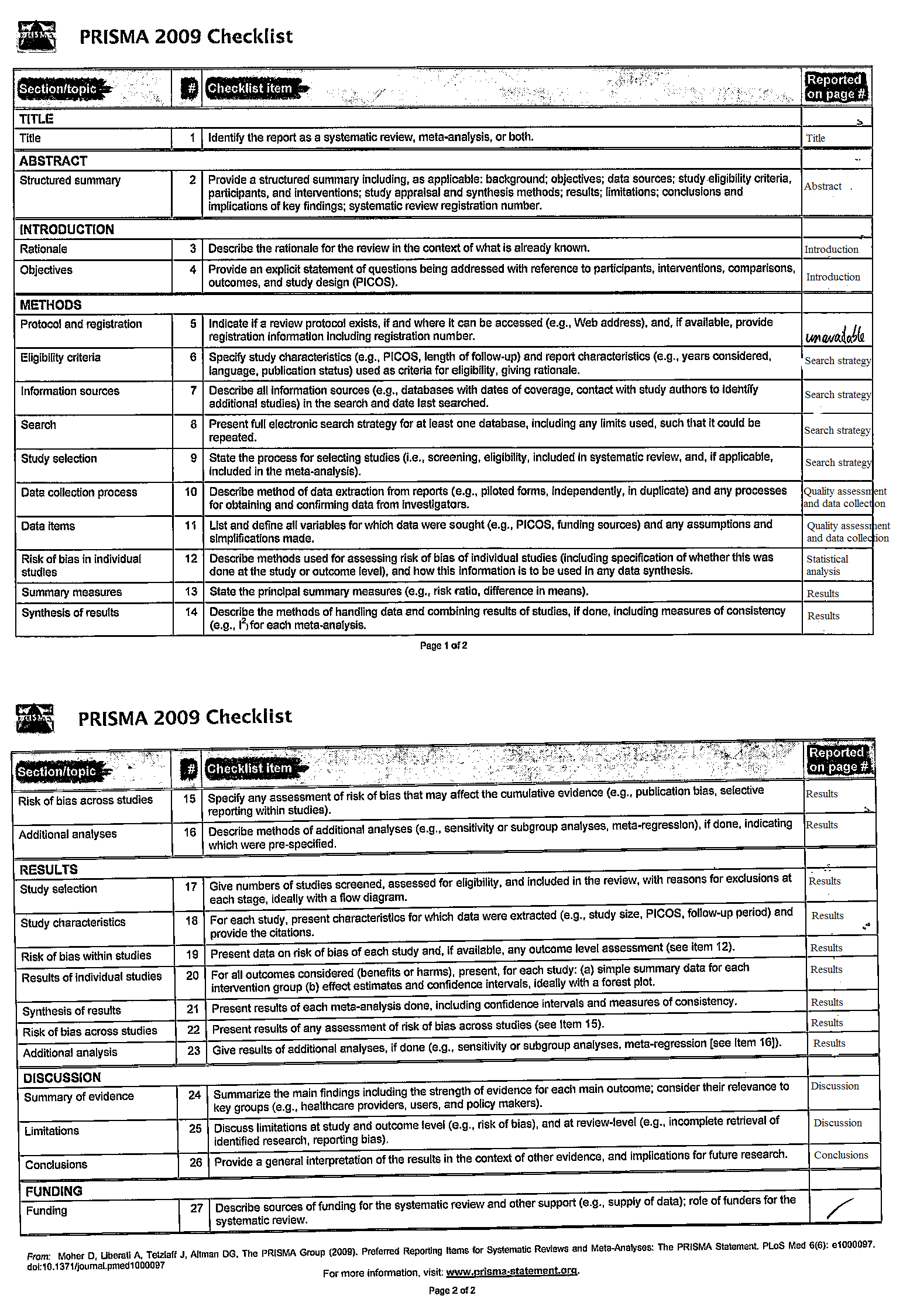

Supplement: Checklist S1 — PRISMA checklist for meta-analysis. (TIF) [file pone.0077901.s001.tif]
